# 3D printing of polypropylene reinforced with hemp fibers: Mechanical, water absorption and morphological properties

**DOI:** 10.1016/j.heliyon.2024.e26617

**Published:** 2024-02-17

**Authors:** Raffay Sultan, Mikael Skrifvars, Pooria Khalili

**Affiliations:** Swedish Centre for Resource recovery, Faculty of Textiles, Engineering and Business, University of Borås, SE-50190, Borås, Sweden

**Keywords:** Additively manufactured, Hemp fiber reinforced polymer, 3D printing, Mechanical properties, Water absorption

## Abstract

The aim of this study was to develop and additively manufacture polypropylene-hemp fiber (PPHF) composites, which were composed of polypropylene (PP) and hemp fibers (HF) in various percentages (5%, 10%, and 20%). The objective was to examine the mechanical properties and water absorption behaviors of extruded PP, conventional filament PP and PPHF composites. The findings of the flexural and tensile tests provided important valuable information. In comparison to the other materials examined, extruded PP had the highest flexural modulus and strength, but filament PP had the lowest mechanical properties. The results showed that the 5% hemp PP composite exhibited the highest tensile strength, and the 20% hemp PP composite showed the highest Young's modulus. These results highlight how crucial hemp fiber content is in modifying the mechanical characteristics of a polymeric material to obtain the material with desirable properties for specific industry requirements.

## 1 Introduction

One of the most popular methods for creating prototypes and producing CAD-designed products is additive manufacturing [1]. It is widely employed in sectors like automotive, composites, healthcare and electronics [2]. It is an extremely practical method for creating inexpensive complex geometries of excellent quality. Along with the popular polymeric materials being used for 3D printing, nowadays more and more fiber-reinforced polymers are also being used for 3D printing. Polymers reinforced with natural fibers are becoming more popular in 3D printing due to the numerous advantages they offer such as altering the mechanical properties of the polymer. In fact, natural fiber-based composites are more recyclable than those reinforced with glass or carbon fibers [3]. The benefits of natural plant fibers over conventional glass fibers were acknowledged in a previous research study, including superior specific strengths and modulus, low density, better energy recovery, decreased skin-related and respiratory irritation along with good biodegradability [4]. Plus, they are more economically viable [4].

Additionally, natural plant fiber-reinforced polymeric composites have certain drawbacks, such as the incompatibility of the hydrophilic natural fibers to bond easily with hydrophobic thermoplastic and thermoset matrices, which necessitates the application of suitable physical and chemical treatments to improve the adhesion between the fiber and the matrix [5]. There are many research studies done in the area of 3D printing but only a few of them are related to reinforcing natural fibers into the material being additively manufactured. LDPE (low-density polyethylene)/Hemp fiber composite's thermal and mechanical characteristics were examined in the study [6] to see if maleic anhydride-grafted hard paraffin wax (MA-g-wax) and oxidized hard paraffin wax (Ox-Wax) might be utilized as compatibilizers. According to the differential scanning calorimetry (DSC) data, adding hemp did not change the crystallization behavior of LDPE on its own, but adding wax to LDPE and having hemp present had a different impact. Both Ox-Wax and MA-g-wax had the same values for tensile strength when added to the composites. Both waxes raised the modulus values of the compatibilized composites but decreased the tensile strengths in both cases, most likely due to the poor adhesion between LDPE and the waxes.

There were many reasons to choose specifically hemp fiber for this study. Today, industrial hemp (Cannabis sativa L.) is produced all over the world, having been farmed for many centuries [7]. It is among the earliest plants that have been utilized to produce food, clothing, and medicine [8]. The only commercially available long natural fibers cultivated in the UK are hemp and flax. Various mechanical techniques are used to process plant stems to obtain the fiber [9]. Due to their higher specific strength and lower cost when compared to conventional reinforcements, hemp and flax fibers have recently been used by automotive manufacturers for producing non-structural components [10].

The research article [11] mentioned less expensive and more environmentally friendly alternative to the costly chemical treatments of fibers in polymer composites is the control of fiber length. Using automatic milling and manual cutting, various lengths of hemp fibers (HF), ranging from 1 to 4 mm, were obtained. A complicated system consisting of a composite made of polypropylene (PP), poly[styrene-b-(ethylene-co-butylene)-b-styrene (SEBS), and HF was used to study the impact of HF length on the properties of the composites. Depending on the original fiber length, a 2, 2.5, or 4 times reduction in HF average length was seen after extrusion and injection molding. Different fiber lengths have a negligible impact on the degradation of composites. The research study [3] aimed to assess the dynamic properties of nonwoven polypropylene (PP) composites reinforced with flax, hemp, kenaf, and glass fibers.

Nowadays, the development of new composite materials is a major focus of fused deposition modelling (FDM) studies. Determining the characteristics of a newly developed material for FDM is essential to improve the quality of the printed objects. The research study [12] intended to determine how fiber affects the mechanical, thermal, and physical properties of thermoplastic composites reinforced with oil palm fiber. The samples underwent mechanical and physical testing, Fourier-transform infrared spectroscopy (FT-IR), thermogravimetric analysis (TGA), differential scanning calorimetry (DSC), and scanning electron microscopy (SEM) to characterize them. In accordance with the findings, the 3 wt% fiber composite had a somewhat greater tensile strength and modulus than pure ABS.

This study was focused on 3D printing PP and its composites, which is always challenging. It alsoexplored different methods to 3D print neat PP and PP filled with hemp fibers (HF). The HF used in this study was derived from hemp sticks, which were waste materials. These hemp sticks were processed to form short fibers for use in 3D printing applications. This approach adds significant value as it utilizes waste materials, contributing to resource recovery and circular economy principles. The objective of this study was to develop composites of Polypropylene-Hemp Fiber (PPHF) by incorporating different weight percentages (5%, 10%, and 20%) of HF into PP. The main focus was on manufacturing PPHF composite materials and analyzing the changes in their mechanical properties with varying percentages of hemp fibers. Additionally, the study aimed to assess the mechanical properties and water absorption behavior of extruded PP, conventional (commercial grade) filament PP, and PPHF composites. The uniqueness of this manuscript lies in the development of a composite material through the reinforcement of different percentages of hemp fibers (5%, 10%, and 20%). What sets this study apart was the utilization of a pellet 3D printer for the additive manufacturing process, which poses inherent challenges, especially when dealing with polypropylene (PP). PP is known for its difficulty in 3D printing, and the incorporation of hemp fibers further complicates the process. Achieving properly printed specimens required a substantial investment of time and effort, making the additive manufacturing of this material a challenging yet noteworthy endeavor.

## 2 Materials and methods

### 2.1 Materials

Polypropylene BE375MO produced by Borealis, with a melt flow index (MFI) of 13g/10min ($230^{0}$/2.16 kg) and a density of 905 kg/ $m^{3}$ was used. Pellets of Polypropylene BE375MO were used to produce extruded PP using extrusion and as a polymer matrix material in PPHF composites. Polypropylene-graft-maleic-anhydride (MAPP), maleic anhydride 8–10 wt% by Sigma-Aldrich having a melting point of 15 $6^{0}$ C was used as a bonding agent between PP and hemp fibers. Hemp stick waste, which was used as the initial source of fibers, was obtained from the University of Borås, the school of textiles which were later processed. Typically, hemp fiber has a length of 8.3–14 mm and a diameter between 17 and 23 μm [13]. Clear PP Filament from the brand Prima Select with a diameter of 2.85 mm was used for the 3D printing of filament PP samples.

### 2.2 Preparation and treatment of hemp fibers (HF)

Hemp originally was in the form of stalks. Then, it was necessary to grind it in order to combine its shives with the polymers. A RETSCH SM 100 grinder was used for this purpose. When stalks were grinded, smaller pieces of hemp fibers were produced still resulting in the clogging of the machinery when too much hemp was added all at once. Then, it became necessary to use modest amounts and regularly remove hemp from the grinder. After the stalks had been grinded, shives of different dimensions were produced. As a result, the grinded material was passed through a series of vibrating sieves with smaller openings of 2, 1, 0.5, and 0.125 mm. The shives with sizes ranging from 1 to 2 mm were chosen. HAVER & BOECKER sieve shakers and NYCANDER sieves were used with vibration operations that lasted 3 min.

Shives were covered in dust as a consequence of the grinding process, which is an undesired impurity in composites (the retted hemp batches were similarly covered with dirt). That is why shives were supposed to be washed before being combined with polymers because of this procedure and sieving. They had been washed in a thinner sieve (so that none of them would pass through the holes) and the washing was stopped when the washing water was sufficiently clear. The application of sodium hydroxide to hemp fibers causes the fiber bundles to separate and their surface roughness to increase [14]. So, some shives were additionally treated by soaking for 5 h in a 4.5 wt percent NaOH solution. The shives were dried in ovens at 80 °C for 24 h after washing or after soaking.

### 2.3 Polypropylene hemp fiber (PPHF) composite material preparation

The initial step for this project was to arrange all the appropriate materials needed for material processing. The main materials were polypropylene pellets, processed hemp fibers and minor amounts of maleic anhydride-grafted PP (MAPP) for better adhesion between PP and fibers. The research study on MAPP [15] mentions that MAPP-treated composites with enough maleic anhydride (MA) graft (%) and molecular weight exhibited increased mechanical and thermal stability. The percentage of MA graft and the MAPP molecular weight had a significant impact on the composite's improved interfacial adhesion, mechanical stability, and thermal stability. The MAPP-treated composite's morphological characteristics indicated efficient bonding and a lack of pulled-out traces from the matrix in the two stages. In addition, a spectral examination of the chemical structure utilizing attenuated total reflectance (FTIR-ATR) was used to confirm the increased interfacial adhesion of the MAPP-treated composites.

The compatibilizer utilized in this study was MAPP, with a fixed quantity of 2% of the total composite material mass across all three composite materials. This fixed amount may not have significantly influenced the properties.

PP pellets were dried using a drying oven for 24 h to remove any moisture from them. HF were washed with warm water in a strainer and then allowed to dry in the oven for 24 h at the temperature $80^{0}C$ before usage. HF were grinded once again to make them even finer before mixing with PP. MAPP was used at room temperature. Once the PP, hemp fibers and MAPP were arranged, three combinations of composite materials were made with 5% fibers, 10 % fibers and 20% fibers along with some additional MAPP added to it for better adhesion. The remaining major material was PP pellets. Material proportion of different composite additives in various mixtures in terms of percentage are shown in Table 1 along with the filament PP and extruded PP:

**Table 1:** Components of PPHF composite material by % mass.

Composite name	Polypropylene (% mass)	Hemp Fibers (% mass)	MAPP (% mass)
5% Hemp PP	95	5	2
10% Hemp PP	90	10	2
20% Hemp PP	80	20	2
Extruded PP	100	0	0
Filament PP	100	0	0

Three types of composite mixtures were made with 5%, 10% and 20% of fibers to analyze later how mechanical properties vary for PP composites with various percentages of HF. Additionally, as mentioned in a previous work [16], the rough structure of raw hemp fibers aids in ensuring appropriate bonding between the fibers and matrix. The mixture was stirred in a plastic beaker with lab spatula for enough time roughly 2 min until the elements were uniformly mixed together with each other. When not mixed in a proper way, the composite material breaks easily [17]. Adhesion between the fiber and matrix is essential for producing natural fiber-reinforced composites with improved mechanical characteristics [18].

### 2.4 Extrusion process

An extruder is one of the best machines that can be used to mix two or more different materials. In this project, a Thermo Fisher Scientific Process 11 Parallel Twin-Screw Extruder with 3 mm die was used for the purpose of extrusion. Firstly, pure PP pellets were extruded with a melting temperature of $215^{0}C$, extruder rpm of 60 and melt flow pump rpm of 30. At first water cooling was given a try to be a potential option to cool the filament before winding on the spool but it was not working. Therefore instead, room temperature cooling on a conveyor belt along with a little bit of air cooling was adopted. After obtaining a spool of extruded PP filament, extrusion of composite materials was started, but the extrusion parameters were changed according to the complexity of the composite material.

**Fig. 1:**
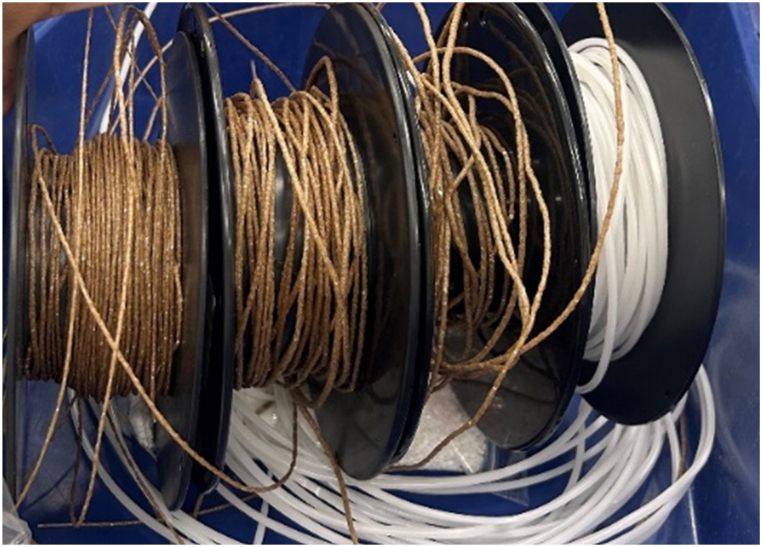
Spools of different materials.

For the extrusion of the composites melt temperature was kept constant, the rpm of the extruder was reduced to 40 as the mixture was quite dense and the rpm of the melt flow pump was reduced to 20 to get a uniformly mixed material. According to the literature, the surface finish of fiber-reinforced polymer composites becomes progressively dull with increasing fiber content [19]. One after one the filaments for each composite were extruded until at least one spool for each filament was obtained. The image of the spools can be seen in Fig. 1.

### 2.5 3D printing process

The filament 3D printers available at the lab demanded a constant diameter of 2.8 mm which was very hard to attain even after several attempts. Another critical issue was that the size of the nozzle of the 3D printing was 0.4 mm where the fibers can cause blockage in the nozzle. Alternatively, it was considered that a pellet 3D printer should be used to 3D print this composite material. For that, all the filament spools were chopped into 2 mm pellets using a pelletizer. Then 3D printing was initiated but the material was not sticking to the bed. To resolve that issue, the bed was taped with PP packaging tape which resulted in better adhesion as PP likes to stick to itself [20,21]. The material started to print well after adjusting various printing parameters such as nozzle temperature ($220^{0}C)$, bed temperature ($90^{0}C$), printing speed (40 mm/s), layer height (0,6 mm), fill angle (90°) and fill pattern (aligned rectilinear). Printing speed was kept low as it can improve the adhesion of the material to the bed [22]. Prusa Slicer was used as a slicing software tool.

Modified Artillery Sidewinder X1 supporting pellet 3D printing was used as a printer. The nozzle used in this process had a diameter of 0.8 mm. This way all the flexural and tensile specimens were produced. After 3D printing, a little bit of post-processing was done by griding irregular areas on the sides of the specimens. The samples produced were quite decent in quality.

**Fig. 2:**
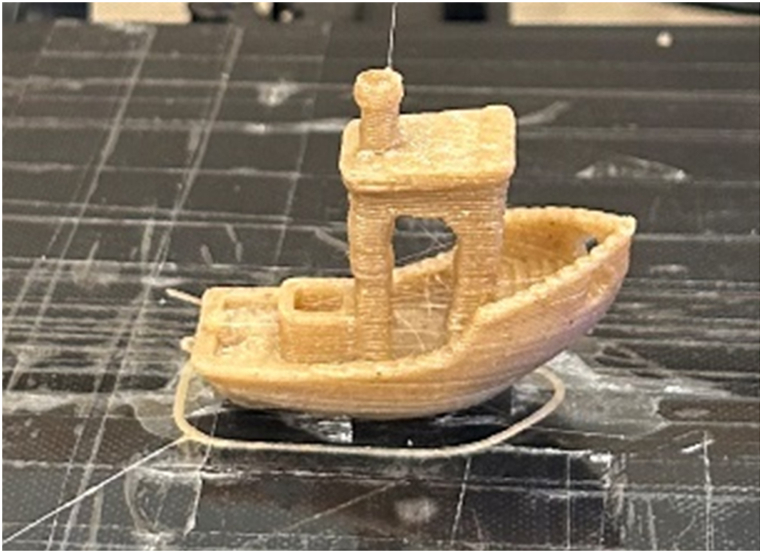
Additively manufactured 3D benchy using PPHF composite material.

After all the specimens of extruded PP and PPHF composites required for mechanical testing were produced and post-processed. A spool of traditional PP filament spool by the brand Prima Select was arranged. It was loaded into filament 3D printer BCN Sigma, in this case, Simplify 3D was used as a slicing software. Using filament PP, the required amounts of flexural and tensile specimens were produced. The main goal was to compare the mechanical properties of extruded PP, filament PP and three fiber-reinforced composite materials to each other. After printing all the specimens, the famous 3D benchy was additively manufactured with all three composite materials containing 5%, 10% and 20% hemp PP to make sure this material is quite practical for obtaining physically complex geometries as 3D prototypes. It printed quite well, as benchy produced from 5% hemp PP can be seen in Fig. 2.

3D benchy is a common 3D printing part used to calibrate printers and adjust the quality of the prints. Using this composite material on that printer, the quality of the obtained benchy was excellent and side curves were also quite fine. This means that this material is quite practical to produce physical parts from complex CAD files.

### 2.6 Characterizations

The research efforts in this study were driven by a systematic and precise approach to characterize the mechanical properties of a variety of materials required for engineering applications. In this project, two types of mechanical testing were done namely flexural testing and tensile testing. Along with that water absorption test was also done to observe the water absorption trend of various materials.

#### 2.6.1 Tensile testing

For tensile testing, the specimen was designed according to the dimensions of ISO 527-4 in CAD software SolidWorks. The specimen had an overall length of 150 mm, the width at the narrow middle section was 10 mm and the thickness was 4 mm. To perform the tensile tests, a Tinius Olsen H10KT universal testing machine equipped with a 5 kN load cell was used. Meticulous compliance with EN ISO 527-4 ensures consistent and reliable testing procedures. For each sample, the distance between the two clamps was set at 115 mm, thus ensuring uniformity of the test configuration. The gauge length was set as 50 mm with an extensometer. To improve the accuracy of strain measurement, a 100R mechanical extensometer has been integrated into the testing process [23].

Tensile testing was performed at a controlled speed of 2 mm per second, allowing precise determination of tensile strength and modulus. Conditioning of the tensile test specimens was done in a climate chamber by placing them at $23^{0}C$ and 50% humidity for 24 h before testing. The diverse range of specimens evaluated in this study included five types, each consisting of five individual specimens. These materials include extruded polypropylene, filament polypropylene and reinforced polypropylene infused with hemp fibers at various concentrations (5%, 10%, and 20%). Attention to detail also extends to the sample preparation stage, where the specimen was meticulously shaped into the shape of a dog bone. Sandpaper was applied to the grip edges of each specimen to eliminate potential sources of error.

#### 2.6.2 Flexural testing

The flexural test specimen was designed according to the standard EN ISO 14125, class 1 discontinuous-fiber reinforced thermoplastics. Dimensions for the rectangular flexural specimen were as per standard with a length of 80 mm, width of 10 mm and thickness of 4 mm. Span length during testing was set as 64 mm.

To perform the flexural tests, a Tinius Olsen H10KT universal testing machine equipped with a 250 N load cell was used. Precise compliance with EN ISO 14125 ensures consistent and reliable testing procedures. A test speed of 5 mm/min was adopted during the testing process. The same five types of materials were used for the purpose of bending testing namely extruded polypropylene, filament polypropylene and reinforced polypropylene infused with hemp fibers at various concentrations (5%, 10%, and 20%). Five specimens of each type were tested, and the average result value was taken into consideration.

#### 2.6.3 Water absorption test

In this test, 3 specimens from each material type were merged in the beakers filled with water and weighed continuously for 19 days to observe the material's response when exposed to water for a longer period. This test was also done with all five types of material. Samples were weighed to get the initial dry mass and then merged in water beakers. Samples were weighed every 24 h and masses were noted every day almost at the same time to see the trend of how the masses of the samples changed by leaving them in water. It was done to study to what extent the material is hydrophobic or hydrophilic.

#### 2.6.4 Digital imaging microscopy

For the broken tensile test specimens of fiber-reinforced materials, the area of breakage from the middle-reduced cross-section was considered for microscopy. One broken sample was taken for each of the PPHF composite (5,10 and 20%). It was cut into tiny rectangular pieces from the breakage area to make it compact enough to fit inside the microscope. Equipment used for this purpose was Nikon Industrial Microscope Eclipse LV100ND attached with a computer to capture and save focused images. Microscopy of the specimens was done to find fiber pull-outs, fiber breakage or porosities.

## 3 Results and discussion

### 3.1 Tensile properties of the materials

The results of the tensile testing for different samples are presented in Table 2. The tensile strength of the extruded PP sample was found to be 20.56 MPa. The same sample showed Young's modulus of 438.53 MPa. Additionally, the sample exhibited an elongation at a yield of 9.37%. In comparison, the filament PP sample had a lower tensile strength of 7.92 MPa. Its Young's modulus was quite low having a value of 139.02 MPa and it showed a significantly higher elongation at a yield of 20.30%. It should be noted that extruded PP was produced from PP pellets, whereas filament PP was a commercial-grade 3D printing filament. The latter was used for comparison with the normal (injection molding) type PP. It was shown that 3D printed PP produced from granules demonstrated better mechanical properties than that of the commercial grade in our study. The 5% hemp PP sample demonstrated a tensile strength of 21.4 MPa and Young's modulus of 382.1 MPa. The elongation at yield for this sample was 11.46%. Meanwhile, the 10% hemp PP sample exhibited a tensile strength of 14.6 MPa. Its Young's modulus was 574.1 MPa and it showed an elongation at yield of 7.6%. Lastly, the 20% hemp PP sample had a tensile strength of 15.56 MPa. Its Young's modulus was 876.43 MPa. 20% hemp PP exhibited the lowest elongation at yield among the samples with a value of 5.39%. These results provide detailed tensile properties of the different materials tested, highlighting variations in strength, modulus, and elongation at yield that are vital properties for various applications and material selection processes. Stoof et al. [24] investigated the 3D printing of hemp fiber in combination with recycled pre-consumer PP and conducted tensile tests. They observed that the addition of hemp fiber into PP did not alter the tensile strength of the printed composite. However, the modulus experienced a 13% increase when the hemp fiber content was raised from 10 wt% to 20 wt%.

**Table 2:** Tensile testing data.

Tensile Testing Sample	Tensile Strength (MPa)	SD Strength (MPa)	Young's Modulus (MPa)	SD Modulus (MPa)	Elongation at Yield (%)
Extruded PP	20.6	1.91	438.5	59.7	9.3
Filament PP	7.92	0.3	139.0	17.8	20.3
5% Hemp PP	21.4	1.0	382.1	86,5	11.5
10 % Hemp PP	14.6	1.4	574.1	119.3	7.6
20% Hemp PP	15.6	0.6	876.4	61.1	5.3

**Fig. 3:**
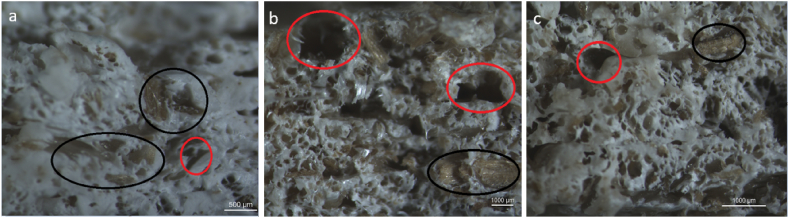
Microscopic images for the cross-sections of broken tensile test specimens of various PPHF composites a) 5% Hemp PP, b) 10% Hemp PP and c) 20% Hemp PP. Black circles show fibers and red circles represent porosities. (For interpretation of the references to colour in this figure legend, the reader is referred to the Web version of this article.)

Later microscopy was done from one of the broken tensile specimens of each PPHF composite (5,10 and 20%) from the broken cross-section (Fig. 3 (a), (b) and (c). The aim behind microscopy was to examine the presence of fibers inside the PP matrix and to search for any porosities or fiber pullouts present inside the material. Areas marked with black circles outline the presence of fibers and areas marked in red circles represent porosities as shown in Fig. 3.

The data obtained through tensile testing for all five materials is shown in Table 2.

The results appear to be quite accurate as they are quite similar to the results found in a similar research study [20], as the 10% hemp PP and 20% hemp PP additively manufactured samples showed lower tensile strength than pure extruded PP. Whereas 5% hemp PP showed highest tensile strength even higher than the extruded PP. It shows that tensile strength increases when PP is reinforced with lower percentages of hemp fiber. Comparatively, filament PP showed the lowest tensile strength. As supported by the results of the research study [20] showed that Young's modulus was higher for 10% hemp PP and 20% hemp PP than pure extruded PP. 20% hemp PP showed the highest Young's modulus. Whereas 5% hemp PP showed a bit lower Young's modulus than extruded PP. Filament PP illustrated the lowest value for Young's modulus and highest elongation at yield.

### 3.2 Flexural properties of the materials

Table 3 shows the findings of flexural testing for several samples. The mechanical properties of natural fiber-reinforced polymers are highly influenced by parameters such as fiber length, fiber orientation inside the material and interfacial bonding [25,26]. The material with the highest flexural modulus was extruded polypropylene (PP) with a value of 1350 MPa. Contrarily, the flexural modulus of filament PP was found to be the lowest with a value of 207 MPa. Extruded PP showed a flexural strength of 36.4 MPa, which determines a material's capacity to endure bending. Lower bending resistance was demonstrated by filament PP, which had a flexural strength of 8.58 MPa.

**Table 3:** Flexural Testing Data of the neat PPs and hemp based composites.

Flexural Testing Sample	Flexural Modulus (MPa)	SD Modulus (MPa)	Flexural Strength (MPa)	SD Strength (MPa)	Maximum Force/N
Extruded PP	1350	99	36.4	0.8	65.4
Filament PP	207	14	8.58	0.4	14.2
5% Hemp PP	1260	90	29.6	2,4	55.5
10% Hemp PP	941	123	22.8	1.6	44.8
20% Hemp PP	1080	130	25.1	1.1	50

*The addition of hemp fibers had noticeable effects on both flexural modulus and strength when investigating the hemp PP samples. The 5% hemp PP sample had a flexural modulus of* 1260 MPa *and a flexural strength of 29.*6 MPa*. The 10% hemp PP sample had a flexural modulus of* 941 MPa *and a flexural strength of 22.*8 MPa*; it had a moderately lower strength than the extruded PP sample. The 20% hemp PP sample, on the other hand, had a flexural strength of 25.*1 MPa *as well as a flexural modulus of* 1080 MPa*. Cai* et al. [27] *explored the 3D printing capabilities of ramie fiber in conjunction with PP and successfully achieved the 3D printing of this material. They conducted bending tests on samples with varied processing parameters.*

### 3.3 Water absorption behaviors of the neat PPs and hemp based composites

**Fig. 4:**
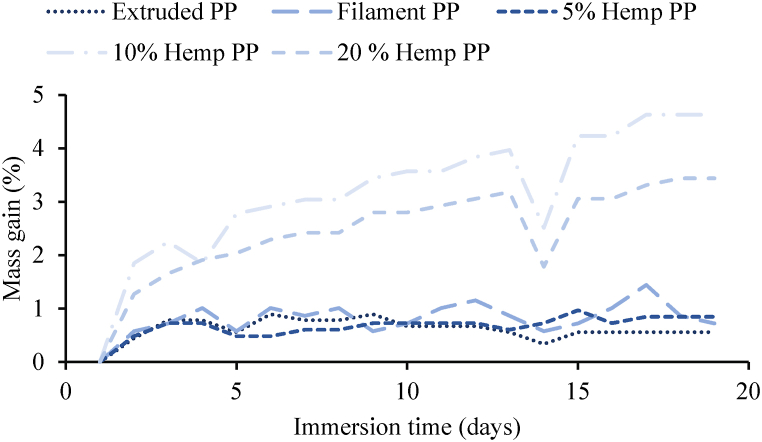
Water absorption behaviors for various materials.

Three specimens from each material were placed inside the water beakers with samples completely immersed in water in each beaker. Their masses were measured every 24 h for almost 19 days to study the water absorption behaviors of these materials and an average value was considered for results. After 19 days, extruded PP showed the least increase in mass of 0.56%. For filament PP it was 0.721%. In the case of 5% hemp PP, it was 0. 5%. 10% and 20% hemp PP showed the highest value for an increase in mass. The distribution of fibers in the polymer matrix is crucial to the overall moisture absorption of composites because natural fibers and the polymer matrix have different moisture absorption characteristics [28]. The results are quite normal as the PPHF composite materials with higher amounts of HF showed a higher increase in mass whereas for others it is even less than 1%. There can be many reasons behind this slight increase in mass for these PPHF composites such as fibers tend to absorb water [29] and moisture absorption increases with an increase in fiber content [30]. Another potential reason could be that 3D-printed samples have pores inside them from where water can get inside. 10% hemp PP gained more mass, this could be due to higher porosity content inside it resulting in higher water absorption as higher porosity is linked to lower relative density [31]. Fig. 3 containing microscopy images also shows that 10% hemp PP sample had more porosity defects than 5% hemp PP. The detailed trend for the water absorption behaviors is shown in Fig. 4.

The percentage of gain in mass was calculated by the mass difference between the samples exposed to the water and the dried samples according to the following equation *(1)* :

(1) $$M\;\left(\%\right)=\left[\left(M_{t}\;-\;M_{0}\right)\;/\;M_{0}\right]\times 100$$

where: $M_{t}$ is the mass (g) of the sample after immersion at time t and $M_{0}$ is the initial mass (g) of dry sample.

### 3.4 Discussion

For the tensile test results, it was evident that as very short fibers were incorporated into the polymer system, the strength and elongation to yield were seen to decrease. This is due to the fact that short fibers cannot transfer the loads to the matrix. The microscope images of hemp fiber composites, in particular 10% hemp PP and 20% hemp PP ones (Fig. 3 (b) and (c)) clearly demonstrate this issue. The presence of pores and defects such as fiber pull-outs can be observed in these two composites. This once again emphasizes the significance of fiber length in the polymer composite systems and its impact on the tensile properties of the resulting composites.

Regarding the bending tests, as the mechanism is polymer dependent (unlike the tensile fracture mechanism), there was not a positive indication of the inclusion of HF in terms of bending strength and modulus in composites when compared to the extruded PP system. In this case, the very short fibers can act as impurities and weaken the resistance of the polymer matrix against bending. This effect becomes more pronounced at higher short fiber loadings, as discovered in this investigation.

The varying tensile and flexural testing outcomes for the various samples can be linked to several factors such as material composition, amount of hemp fibers, processing method, orientation of the fibers and printing quality [25,26]. The cause for the modest variations in test results could be due to a variety of factors. Few research studies have examined how the printing process affects the mechanical properties of 3D-printed PP composites in the literature. A research study [32] examined how printing parameters affected the mechanical characteristics of additively manufactured PP composites. The findings demonstrated that the mechanical characteristics of printed composites were significantly influenced by both printing orientation and infill density. Infill density plays a crucial role in making the part stronger; therefore 100% infill was used while printing these samples. A different research article [33] examined the impact of three printing parameters namely extrusion temperature, printing speed, and layer thickness on the mechanical characteristics of a filled polypropylene. Another crucial factor is the quality of 3D printing as it determines the amount of bonding between the layers. The mechanical characteristics, durability, and recyclability of such composites are also influenced by the quality of the interface between the natural fibers and the polymeric matrix [29]. If the layers are strongly bonded to each other, it makes the specimen stronger. On the other hand, minor porosity defects inherited inside the specimen due to the printing process can weaken it [34] and serve as potential sites for crack initiation.

## 4 Conclusions

This study aimed to reinforce hemp fibers (HF) with percentages (5,10 and 20%) into PP, to make various PPHF composite materials. The mechanical properties of extruded PP in its original form and traditional PP filament available for 3D printing were compared to those of PPHF composites. Five various materials were 3D printed extruded PP, filament PP and PPHF composites with (5,10 and 20%) hemp fibers inside them. After studying their mechanical characteristics and water absorption patterns, a conclusion was reached. In summary, the tensile and flexural testing results provide valuable insights into the mechanical properties of Polypropylene (PP) and PPHF composites. The extruded PP showed the highest flexural modulus and flexural strength as flexural properties are mostly polymer dependent.

Filament PP showed the lowest flexural and tensile properties which means all the PPHF composites had better mechanical properties than that of traditional PP filament. 5% hemp PP showed the highest tensile strength. 20% hemp PP had the highest Young's modulus amongst all specimens. Whereas 10% hemp PP exhibited the second highest value for Young's modulus. These results highlight how crucial hemp fiber content is in producing materials with desirable properties. Hemp fibers and PP work well together to provide sustainable, high-performance materials with specific mechanical characteristics for a range of industrial applications. The strength of this study lied in successfully 3D printing the intricate PP composite material and conducting detailed mechanical testing. However, the study was limited to exploring the mechanical properties of PPHF composites with hemp fibers at percentages of 5%, 10%, and 20%. Future research endeavors could explore additional percentages of hemp fiber reinforcements and aim for even higher print quality by adjusting printing parameters. These composites can be further improved for specific industry needs through further research.

## Ethics statement

The present study, focusing solely on the 3D printing process without any potential harm to humans, animals, or other entities, did not require review or approval by an ethics committee.

## Data availability

Data related to this study has been placed in a repository within our research group for prospective use. Upon request, the data will be accessible to external researchers.

## CRediT authorship contribution statement

**Raffay Sultan:** Writing – original draft, Methodology, Formal analysis, Data curation. **Mikael Skrifvars:** Visualization, Supervision, Resources, Formal analysis. **Pooria Khalili:** Writing – review & editing, Validation, Funding acquisition, Formal analysis, Conceptualization.

## Declaration of competing interest

The authors declare that they have no known competing financial interests or personal relationships that could have appeared to influence the work reported in this paper.
